# Supplementary material for: Seasonal Variations and Interspecific Differences in Metabolomes of Freshwater Fish Tissues: Quantitative Metabolomic Profiles of Lenses and Gills
Source: Metabolites. 2019 Nov 2;9(11):264. doi: 10.3390/metabo9110264 (PMC6918250; doi:10.3390/metabo9110264)
Supplement: Supplementary file 1 [file metabolites-09-00264-s001.zip › Supplementary Information.pdf]

Supplementary Information

for

Quantitative metabolomic profiling of lenses and gills of freshwater fish *Sander lucioperca* and *Rutilus rutilus lacustris*

Yuri P. Tsentalovich[1,2], Vadim V. Yanshole[1,2], Lyudmila V. Yanshole[1,2], Ekaterina A. Zelentsova[1,2], Arsenty D. Melnikov[1,2], Renad Z. Sagdeev[1,2]

[1]International Tomography Center SB RAS, Institutskaya 3a, Novosibirsk 630090, Russia
[2]Novosibirsk State University, Pirogova 2, Novosibirsk 630090, Russia
*e-mail: yura@tomo.nsc.ru

Table of content

Supplementary Table S1. Characterization of fish lenses and gills used in this study.

Supplementary Figure S1. NMR spectrum of NMR spectrum of serine phosphoethanolamine (Ser-PETA).

Supplementary Figure S2. NMR spectrum of threonine phosphoethanolamine (Thr-PETA).

Supplementary Figure S3. ESI-Q-TOF mass-spectrum of CID fragments (MS/MS) of isolated parent ion with m/z 229.0586 (Ser-PETA).

Supplementary Figure S4. ESI-Q-TOF mass-spectrum of CID fragments (MS/MS) of isolated parent ion with m/z 243.0737 (Thr-PETA).

Supplementary Table S1. Characterization of fish lenses and gills used in this study.

Sample Number	Fish Type	Date of catching	State of water area	Lens weight, mg	Gills weight, mg
Group 1					
SA1	*S. lucioperca*	10.10.2017	Open water	158	-
SA2	*S. lucioperca*	10.10.2017	Open water	154	-
SA3	*S. lucioperca*	10.10.2017	Open water	94	-
SA4	*S. lucioperca*	10.10.2017	Open water	75	-
SA5	*S. lucioperca*	10.10.2017	Open water	135	-
SA6	*S. lucioperca*	10.10.2017	Open water	94	-
SA7	*S. lucioperca*	10.10.2017	Open water	79	-
SA8	*S. lucioperca*	10.10.2017	Open water	140	-
Group 2					
SW1	*S. lucioperca*	02.02.2018	Ice	98	92
SW2	*S. lucioperca*	02.02.2018	Ice	79	116
SW3	*S. lucioperca*	02.02.2018	Ice	78	85
SW4	*S. lucioperca*	02.02.2018	Ice	109	89
SW5	*S. lucioperca*	02.02.2018	Ice	114	124
SW6	*S. lucioperca*	02.02.2018	Ice	80	63
SW7	*S. lucioperca*	02.02.2018	Ice	73	-
Group 3					
RA1	*R. rutilus lacustris*	28.11.2017	Ice	47	-
RA2	*R. rutilus lacustris*	28.11.2017	Ice	33	-
RA3	*R. rutilus lacustris*	28.11.2017	Ice	49	-
RA4	*R. rutilus lacustris*	28.11.2017	Ice	42	-
RA5	*R. rutilus lacustris*	28.11.2017	Ice	35	-
RA6	*R. rutilus lacustris*	28.11.2017	Ice	32	-
RA7	*R. rutilus lacustris*	28.11.2017	Ice	49	-
RA8	*R. rutilus lacustris*	28.11.2017	Ice	35	-
RA9	*R. rutilus lacustris*	28.11.2017	Ice	38	-
RA10	*R. rutilus lacustris*	28.11.2017	Ice	43	-
Group 4					
RW1	*R. rutilus lacustris*	09.02.2018	Ice	35	82
RW2	*R. rutilus lacustris*	09.02.2018	Ice	35	84
RW3	*R. rutilus lacustris*	09.02.2018	Ice	42	135
RW4	*R. rutilus lacustris*	09.02.2018	Ice	34	220
RW5	*R. rutilus lacustris*	09.02.2018	Ice	34	60
RW6	*R. rutilus lacustris*	09.02.2018	Ice	-	130
RW7	*R. rutilus lacustris*	09.02.2018	Ice	-	103
RW8	*R. rutilus lacustris*	09.02.2018	Ice	-	72

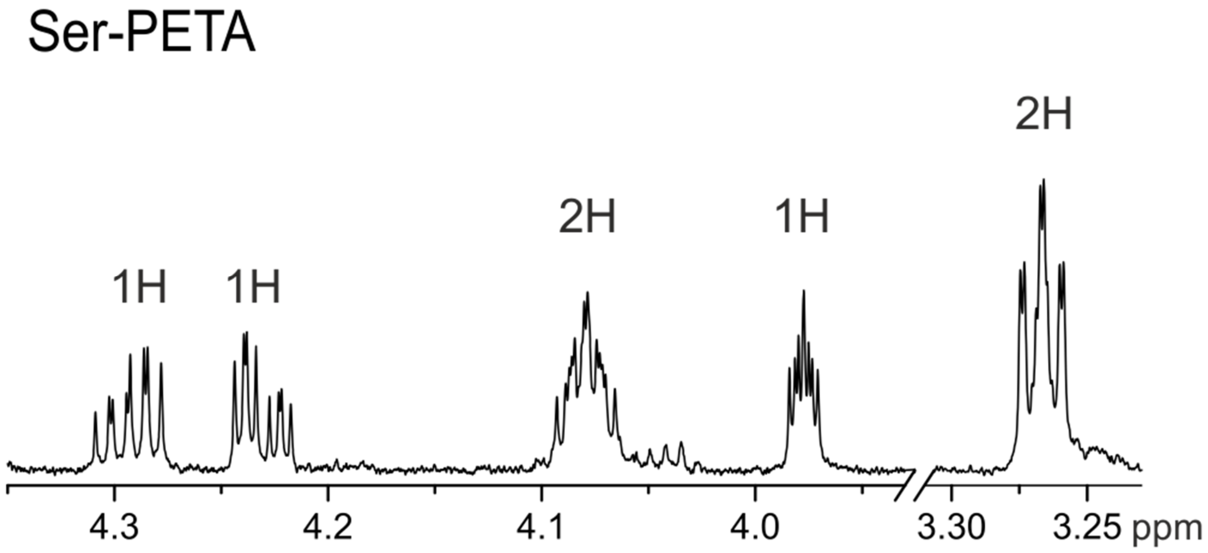

Supplementary Figure S1. NMR spectrum of serine phosphoethanolamine (Ser-PETA). ^1H NMR (700 MHz, D_2O): 3.267 (2H, m, CH_2); 3.978 (1H, m, CH); 4.079 (2H, m, CH_2); 4.23 (1H, m,CH_2); 4.29 (1H, m,CH_2).

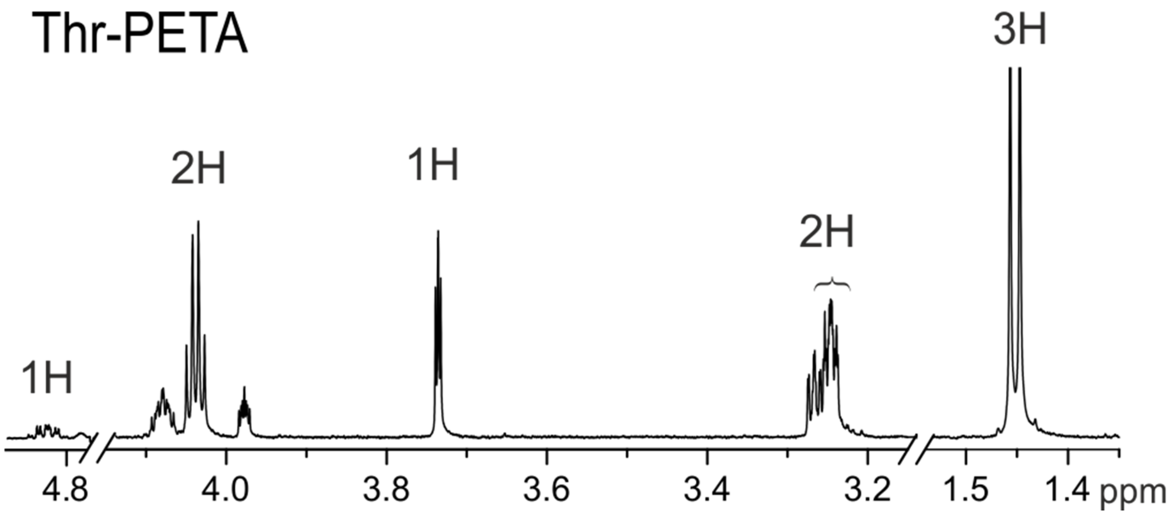

Supplementary Figure S2. NMR spectrum of threonine phosphoethanolamine (Thr-PETA). ^1H NMR (700 MHz, D_2O): 1.452 (3H, d, J = 6.7 Hz, CH_3); 3.247 (2H, m, CH_2); 3.736 (1H, dd, J = 2.2, 2.5 Hz, CH); 4.039 (2H, q, J=5.3 Hz, CH_2); 4.820 (1H, m, CH). The intensity of the signal at 4.82 ppm is attenuated due to its proximity to water resonance suppressed by RF irradiation.

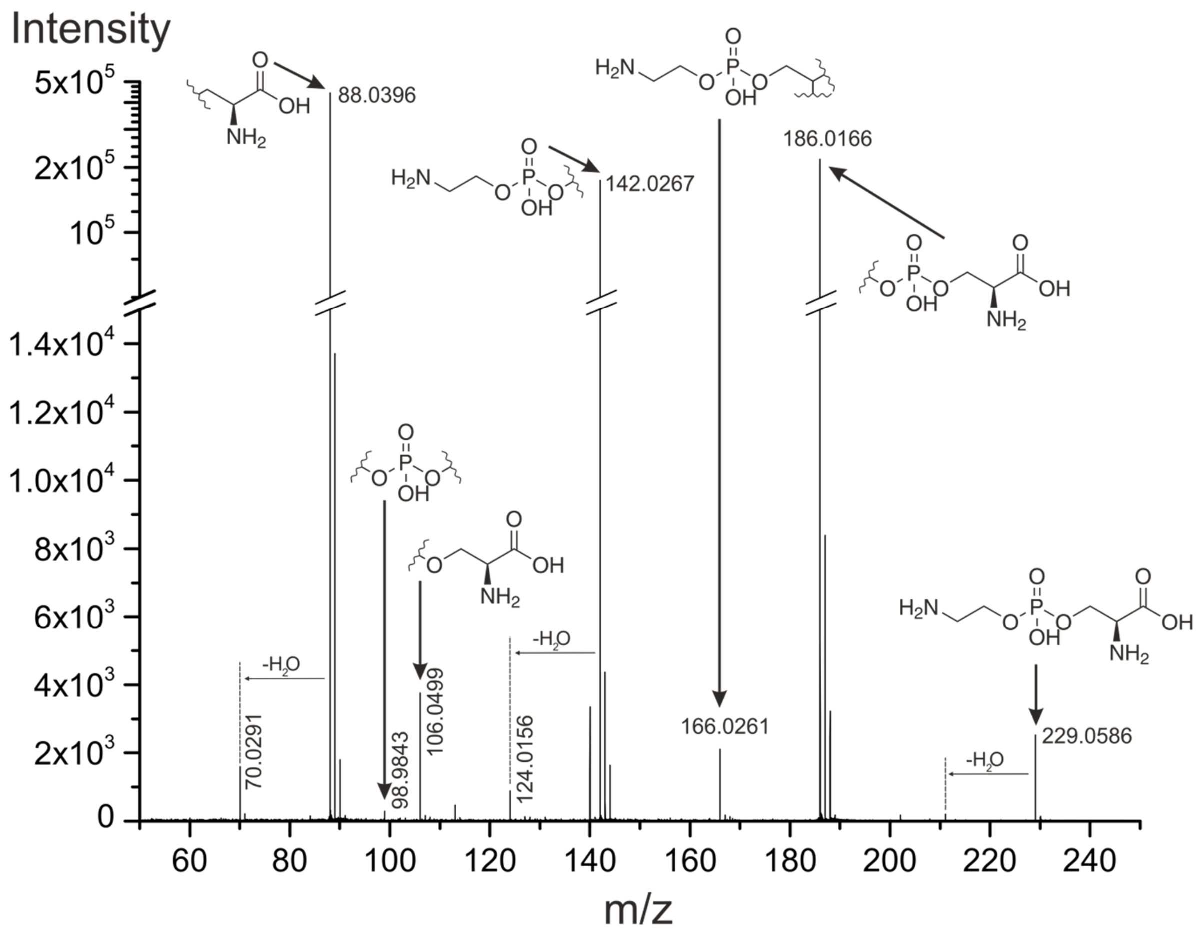

Supplementary Figure S3. ESI-Q-TOF mass-spectrum of CID fragments (MS/MS) of isolated parent ion with m/z 229.0586 (Ser-PETA). Collision energy 35eV, positive ion mode. Wavy lines indicate the positions of the bond cleavage.

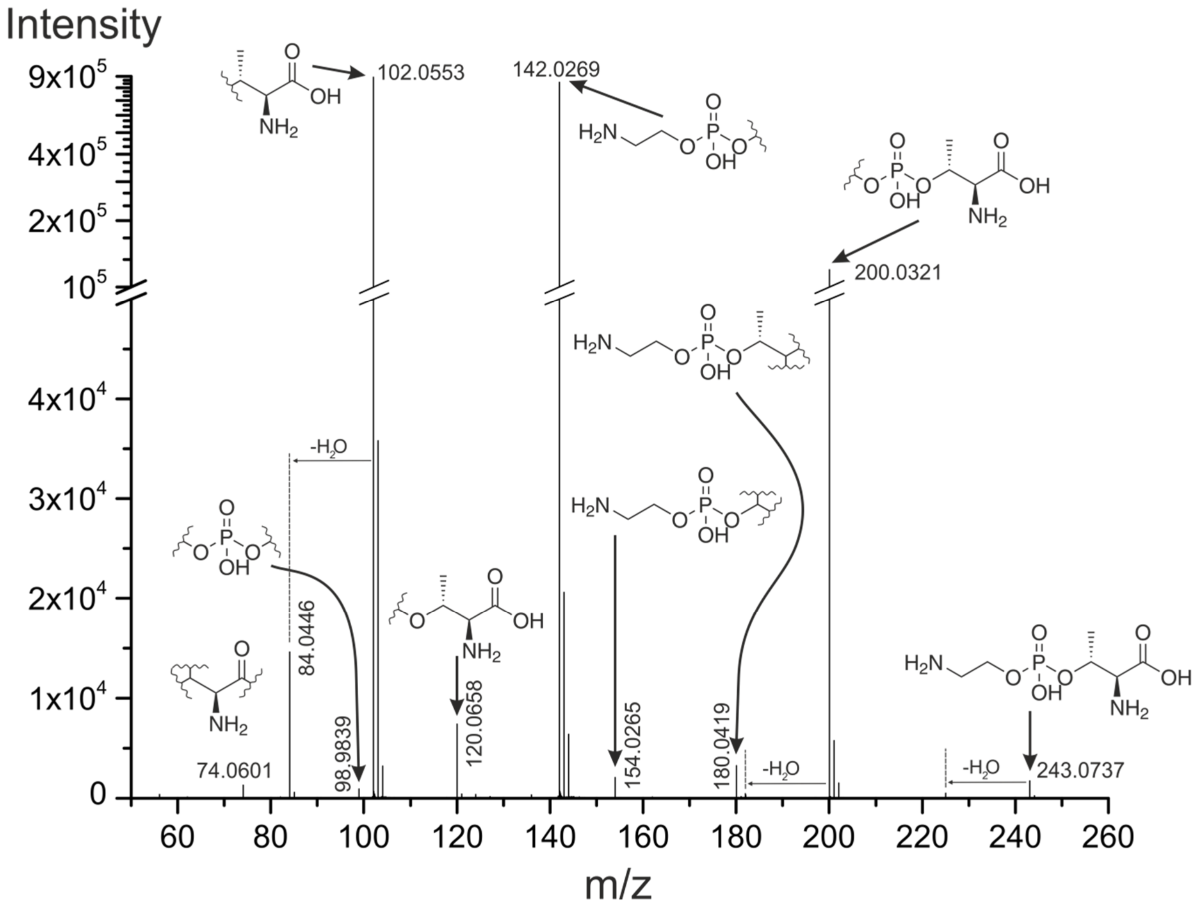

Supplementary Figure S4. ESI-Q-TOF mass-spectrum of CID fragments (MS/MS) of isolated parent ion with m/z 243.0737 (Thr-PETA). Collision energy 35eV, positive ion mode. Wavy lines indicate the positions of the bond cleavage.
